# Supplementary material for: Livestock as vectors of organic matter and nutrient loading in aquatic ecosystems in African savannas
Source: PLoS One. 2021 Sep 8;16(9):e0257076. doi: 10.1371/journal.pone.0257076 (PMC8425544; doi:10.1371/journal.pone.0257076)
Supplement: Supplementary file 3 [file pone.0257076.s001.docx]

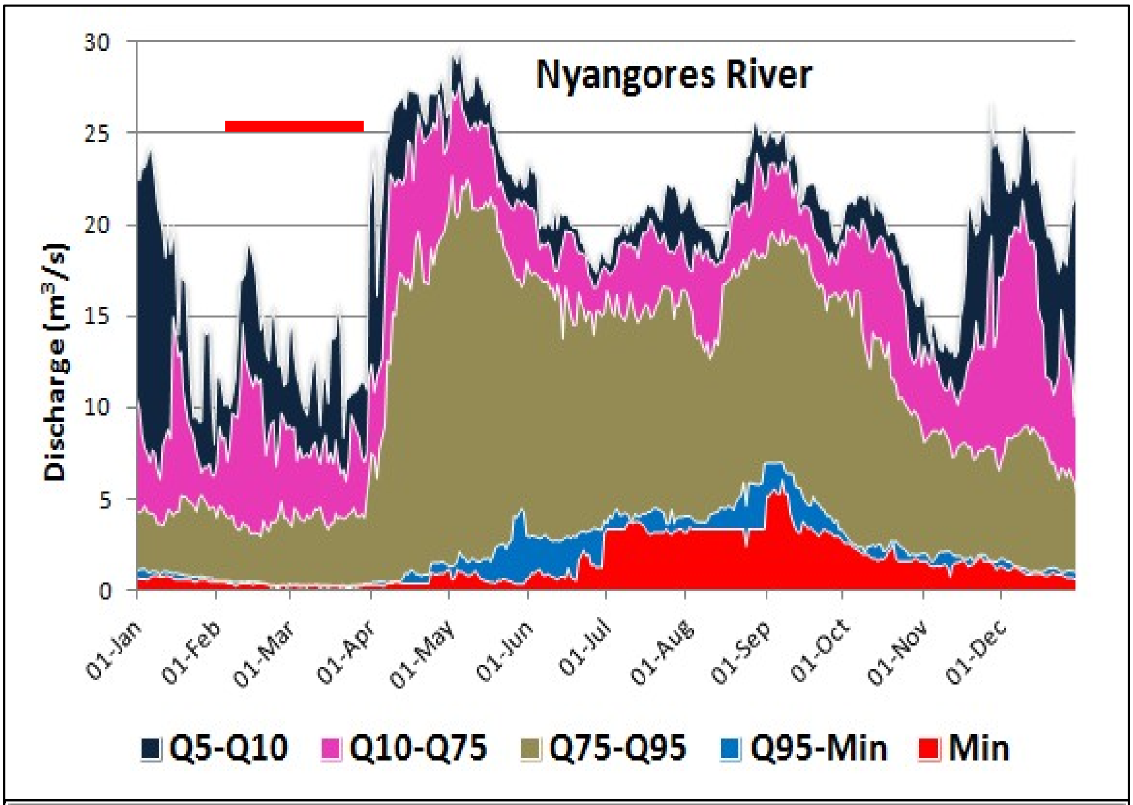
Nyangores River
Discharge (m³/s)
30
25
20
15
10
5
0
01-Jan
01-Feb
01-Mar
01-Apr
01-May
01-Jun
01-Jul
01-Aug
01-Sep
01-Oct
01-Nov
01-Dec
Q5-Q10
Q10-Q75
Q75-Q95
Q95-Min
Min

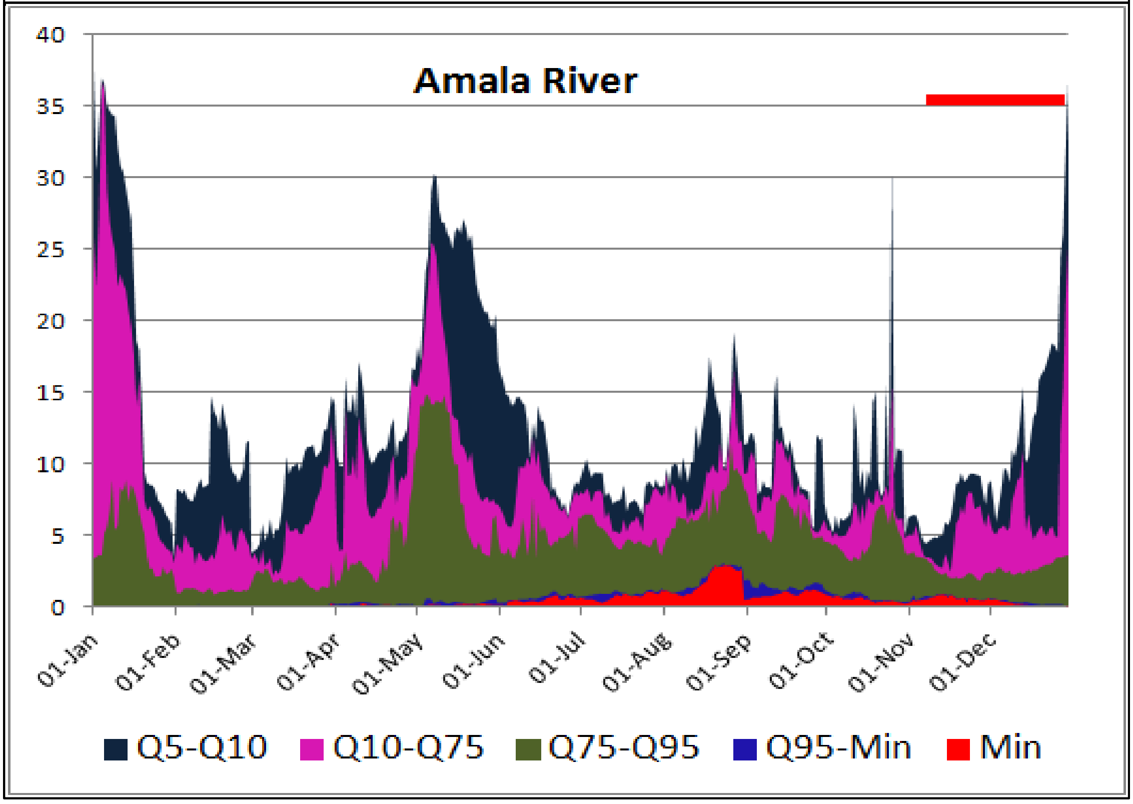
Amala River
40
35
30
25
20
15
10
5
0
01-Jan
01-Feb
01-Mar
01-Apr
01-May
01-Jun
01-Jul
01-Aug
01-Sep
01-Oct
01-Nov
01-Dec
Q5-Q10
Q10-Q75
Q75-Q95
Q95-Min
Min
